# *ANK1* is up-regulated in laser captured microglia in Alzheimer’s brain; the importance of addressing cellular heterogeneity

**DOI:** 10.1371/journal.pone.0177814

**Published:** 2017-07-12

**Authors:** Diego Mastroeni, Shobana Sekar, Jennifer Nolz, Elaine Delvaux, Katie Lunnon, Jonathan Mill, Winnie S. Liang, Paul D. Coleman

**Affiliations:** 1 Biodesign, ASU-Banner Biodesign Neurodegenerative Disease Research Center, and School of Life Sciences, Arizona State University, Tempe, AZ, United States of America; 2 Banner Sun Health Research Institute, 10515 West Santa Fe Drive, Sun City, AZ, United States of America; 3 Translational Genomics Institute, 445 North Fifth Street, Phoenix, AZ, United States of America; 4 University of Exeter Medical School, RILD, University of Exeter, Devon, United Kingdom; 5 Institute of Psychiatry, Psychology and Neuroscience, King's College London, De Crespigny Park, London, United Kingdom; Nathan S Kline Institute, UNITED STATES

## Abstract

Recent epigenetic association studies have identified a new gene, *ANK1*, in the pathogenesis of Alzheimer’s disease (AD). Although strong associations were observed, brain homogenates were used to generate the data, introducing complications because of the range of cell types analyzed. In order to address the issue of cellular heterogeneity in homogenate samples we isolated microglial, astrocytes and neurons by laser capture microdissection from CA1 of hippocampus in the same individuals with a clinical and pathological diagnosis of AD and matched control cases. Using this unique RNAseq data set, we show that in the hippocampus, *ANK1* is significantly (*p<0*.*0001*) up-regulated 4-fold in AD microglia, but not in neurons or astrocytes from the same individuals. These data provide evidence that microglia are the source of *ANK1* differential expression previously identified in homogenate samples in AD.

## Introduction

Alzheimer’s disease (AD) is a multi-factorial neurological disorder classically characterized by amyloid plaques[1] and neurofibrillary tangles[2]. AD also features many signs of chronic inflammation[3, 4]. Microglia, resident immune cells within the central nervous system, are key regulators of the inflammatory cascade[5], which has been proposed as an early event in AD[6, 7]. It has been known for decades that microglia have the ability to release neuro-toxic inflammatory factors, which have prompted hundreds of studies to address a causal mechanism[8]. Although may genetic association studies have identified potential glial-associated alterations (e.g. TREM2) [9], none have been absolute in predicting disease. Recent studies have reached beyond genetic variation, but instead looked towards epigenetic changes as a mechanism by which environment can interact with genome.

As such, recent epigenetic-wide association studies (EWAS) have identified a class of genes associated with AD risk. Ankyrin repeat domain-containing proteins are plasma bound membrane proteins, which modulate interactions between cytoskeletal and membrane proteins[10]. A specific ankyrin repeat domain-containing protein, *ANK1*, has been a source of great interest in the field of AD[11–13] and type 2 diabetes[14, 15], a recognized risk factor for AD[16]. Two independent EWAS have identified methylation changes in the *ANK1* gene that are associated with AD[11, 12]. These studies indicate that epigenetic mechanisms (e.g. DNA methylation) and the rise of age-dependent inflammatory conditions are major risk factors for AD, and *ANK1* may be the link between the two[12]. However, these studies which draw attention to *ANK1* as a potentially critical molecule in the pathophysiology of AD were conducted on homogenates brain samples, which leaves unresolved the identity of the cells impacted by DNA hypermethylation in AD. Here, we provide evidence that microglia are the source of *ANK1* differential expression in the AD brain.

## Methods

### Ethics statement

Written informed consent for autopsy was obtained in compliance with institutional guidelines of Banner Sun Health Research Institute. Banner Sun Health Research Institute Review Board approved this study including recruitment, enrollment, and autopsy procedures. Individual person(s) and their respective next-of-kin consented to brain autopsy for the purpose of research analysis as participants in the Banner Sun Health Research Institute brain and body donation program. The human brain tissue used in this manuscript was from routine existing autopsies, which fully qualifies for 4C exemption by NIH guidelines. In addition, samples were analyzed anonymously (e.g. sample numbers) throughout the experimental process.

### Laser capture microdissection of microglia and RNA sequencing

#### Tissue collection

Frozen unfixed tissue containing CA1 of hippocampus were collected at Sun Health Research Institute (Alzheimer's Disease Centers) from clinically and neuropathologically classified late-onset AD-afflicted individuals with a mean age of 74.6 ± 6.8 years; healthy elderly controls with a mean age of 73.6 ± 6 years; and Parkinson’s disease cases with a mean age of 74.6 ± 15.6 years. Detailed sample information, including post mortem interval (PMI) can be found in Table 1.

**Table 1:** Clinical and pathological patient demographics.

Gender	Clinical and Pathological Diagnosis	Expired Age	PMI	APOE	Braak NFT Stage	RIN Values Whole Tissue
Male	ND	93	3	3/3	I	6.9
Male	ND	73	2.5	2/3	II	7
Male	ND	89	5.5	3/3	II	7.8
Male	ND	79	3	3/3	II	7
Male	ND	53	3.66	3/3	I	7.1
Male	ND	61	2.33	3/3	I	7
Male	AD	75	2	3/4	V	7.3
Male	AD	70	2.33	3/4	V	6.7
Male	AD	76	4	4/4	V	7.1
Male	AD	64	2.33	3/3	VI	7.2
Male	AD	82	2.95	3/3	V	7.3
Male	AD	75	3.83	3/4	V	7.1
Male	PD	69	2.25	3/4	II	7
Male	PD	72	3.5	2/3	II	7.2
Male	PD	70	2.33	3/3	II	7.8
Male	PD	69	3.37	3/3	II	7.5
Male	PD	78	2.75	3/3	II	7.1
Male	PD	86	3	3/3	III	6.9

#### Immunohistochemistry for laser capture

Frozen brain tissue sections (10μm) were mounted onto PEN slides and fixed in ice cold acetone/ethanol for 10 min on ice. Sections were washed in ice cold 1X Phosphate buffered saline (PBS), blocked in 1% hydrogen peroxide for 2 minutes, followed by 3 quick submersions in ice cold 1X PBS. Sections were then placed in 1:500 dilution of primary antibody in 1X PBS supplemented with 50U RNAseOUT (Invitrogen) for 10 minutes at room temperature. After the incubation, sections were washed three times in 1X PBS and incubated in avidin(1:300)-biotin(1:300) complex (Vector) in 1X PBS for 10 minutes at room temperature (RT). Sections were washed three times in 50mM Tris buffer and immersed in 3.3’-diaminobenzidine **(**DAB) solution (9.3 ml 50mM Tris; 200 μl DAB (5mg/ml); 500 μl saturated nickel; and 4 μl of 1% H_2_0_2_) for 5 minutes, followed by two quick rinses in 50mM Tris to stop the reaction.

#### Laser capture of LN3-positive microglia

LN3 antibody reacts with MHCII microglia. These microglia are said to be antigen presenting (e.g. activated, M1 phase, but not phagocytic M2). Immediately after detection of LN3-immunoreactivity, sections were dipped in 100% ethanol and loaded onto a Leica AS-LMD laser capture microscope. After objective calibration, 600 LN3-positive cells were captured using 20X magnification. Individual microglial cells were cut and dropped into an inverted microcentrifuge cap containing 50μl buffer RLT (RNeasy Micro Kit—Qiagen) and 1% β-Mercaptoethanol. Detailed schematic of laser capture can be seen in (S1 Fig).

#### Laser capture of GFAP-positive Astrocytes

Glial fibrillary acidic protein is an intermediate filament protein that is expressed by astrocytes. Immediately after detection of GFAP-immunoreactivity, sections were dipped in 100% ethanol and loaded onto a Leica AS-LMD laser capture microscope. After objective calibration, 600 GFAP-positive cells were captured using 20X magnification. Individual astrocytes were cut and dropped into an inverted microcentrifuge cap containing 50 μl buffer RLT (RNeasy Micro Kit—Qiagen) and 1% β-Mercaptoethanol.

#### Laser capture of pyramidal neurons

Brain sections were stained with 1% neutral red (Fisher Scientific); pyramidal neurons were identified by their characteristic size, shape, and location. Immediately after staining sections were dipped in 100% ethanol and loaded onto a Leica AS-LMD laser capture microscope. After objective calibration, 300 pyramidal neurons were captured using 20X magnification. Individual neurons were cut and dropped into an inverted microcentrifuge cap containing 50 μl buffer RLT (RNeasy Micro Kit—Qiagen) and 1% β-Mercaptoethanol.

#### RNAseq library preparation

Total RNA was used to generate whole transcriptome libraries for RNA sequencing. Using the Nugen Ovation RNA-Seq System v2, total RNA was used to generate double stranded cDNA, which is subsequently amplified using Nugen’s SPIA linear amplification process. Amplified products were cleaned using the QIAquick PCR Purification Kit (Qiagen) and quantitated using Quant-iT Picogreen (Invitrogen). 1 μg of amplified cDNA was fragmented on the Covaris E210 to a target size of 300bp. Kapa Biosystems’ Library Preparation Kit and Illumina adapters and sequencing primers were used for preparing libraries from amplified cDNA. Final libraries were quantified using the Agilent Bioanalyzer and Life Technologies/Invitrogen Qubit.

#### Paired end sequencing

Libraries with a 1% phiX spike-in were used to generate clusters on HiSeq Paired End v3 flowcells on the Illumina cBot using Illumina’s TruSeq PE Cluster Kit v3. Clustered flowcells were sequenced by synthesis on the Illumina HiSeq 2000 using paired-end technology and Illumina’s 200 cycle TruSeq SBS Kit, extending to 83 bp for each of two reads and a 7bp index read. Approximately 250–300 Gigabases (Gb) of reads were generated per flowcell based on Illumina’s published throughput. Quality Control (QC) Steps: During library preparation, electrophoretically separated fragmented cDNA was analyzed on a TAE gel to verify fragmentation. Final libraries were run on a Bioanalyzer to verify sizes and quantitate libraries prior to sequencing. During and following sequencing, we evaluated the total amount of Q30 data generated per library, total number of reads generated, and the total number of mappable reads.

#### Data analysis

Following completion of sequencing, we initiated an automated in-house pipeline for next generation sequencing (NGS) analysis through our laboratory database. From within this database, we have automated the generation of pipeline configuration files for NGS data processing, which generates pipeline analysis without manual intervention. Our analysis pipeline produces standard platform independent formats and a summary of sequencing analysis. Each software tool, including those internally developed, has been validated on multiple simulated and model data sets to characterize each tool’s strengths and biases. At each step within the pipeline, statistics files were created to ensure that all processes have completed and files are uncorrupted. Early within the pipeline, several additional checks are invoked including estimates of overall coverage on a library, evenness of a library, quality of bases, and contamination checks. This pipeline is built around (1) standardized file formats; (2) multiple quality control checks; (3) automated processing; (4) scheduled releases of sequence data, sequencing alignments, and variant calls; and (5) centralized primary data processing. Reads first went through a series of QC steps, quantifying for biases at any given base, and were then parsed by barcode into independent FASTQ files. FASTQ alignments were aligned with TopHat [17, 18]. Alignment produced single BAM files containing both aligned and unaligned data.

#### Statistical analysis

TopHat and Cufflinks were used to assemble reads into transcripts, and HTSeq was used to generate counts data from Cufflinks’ output. DESeq2 calculates normalized read counts for each gene/transcript and uses a generalized linear model to evaluate differential expression between sample groups while accounting for biological variance. DESeq2 uses a Wald statistic as a measure of significance, and the Benjamini and Hochberg False Discovery Rate (FDR) were used for multiple testing corrections. Genes with an adjusted, or corrected, P < 0.05 were evaluated. Variability in the number of reads was accounted for during normalization of read counts. Therefore, we collected data from samples which gave us at least 50 million reads; samples with lower than 50 million reads were omitted. Intronic sequences were analyzed using an unbiased approach to evaluate changes in expression of all RNAs but not small RNAs. Final libraries were run through the Bioanalyzer to verify sizes, and to quantitate libraries prior to sequencing. During and following sequencing, we evaluated the total amount of Q30 data generated per library, total number of reads generated, and the total number of mappable reads. RNA sequencing data can be accessed at National Institute on Aging Genetics of Alzheimer's Disease Data Storage Site, accession number NG00057.

### Hippocampal homogenates and affymetrix arrays

#### Tissue collection

As previously published[19, 20], frozen unfixed tissue was obtained from 26 non-AD controls (age 69–99 years), and 18 Alzheimer’s disease cases (age 74–95 years) (from seven nationally recognized brain banks. Refer to references[19, 20] for detailed sample information. Total RNA was extracted from hippocampus as described previously^19^. Forty-four hippocampal tissue samples were individually hybridized to Affymetrix HgU133 plus 2.0 arrays.

We preferentially selected those probe sets that were most specific, such that they were annotated with the smallest number of Ensembl gene IDs. After applying this criterion, if there remained multiple probes for any one gene, we excluded those probe sets expected to hybridize with targets in a non-specific fashion (i.e., those with “_x_” in the Affymetrix identifier). If there still remained multiple probe sets for a given gene, we took the mean of both probe sets.

#### Statistical analysis

Select genes were investigated for statistical significance (*p* < 0.01) in aging/ND (*n* = 26, age 69–99, mean age 82 ± 9.8 years) versus AD (*n* = 26, age 69–99, mean age 82 ± 9.8 years All genes that did not meet the 50% present call threshold were removed by Genespring G 7.3.1 Expression Analysis software. A two-tailed paired t-test, assuming equal variance (using multiple testing corrections, by Benjamini and Hochberg False Discovery Rate), was applied to locate genes that were significant in differentiating expression between AD and ND cases.

## Results

### *ANK1* expression levels are increased in AD microglia, but not in AD Astrocytes or AD neurons from the same individuals

Current data shows a strong significant hypermethylation of *ANK1 in* AD cortex [11, 12], however no data exist on which classes of cell(s) are associated with these changes. In order to connect the observed changes in *ANK1* to a specific cell type, we mined our previously published homogenate mRNA data [19, 20], and compared these findings to RNA sequencing data we generated from microglia, astrocytes and neurons from the same subjects.

**Fig 1:**
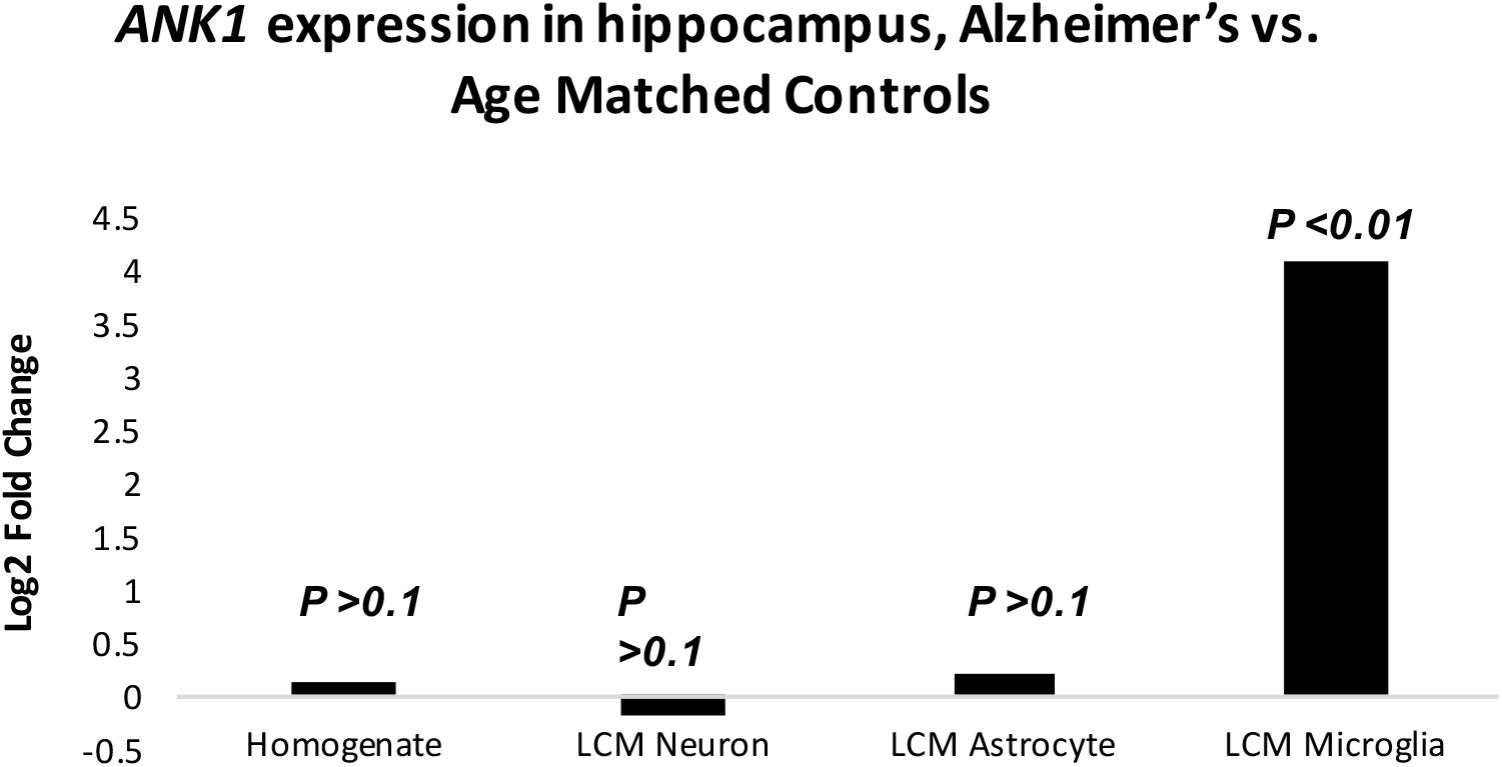
*ANK1* mRNA expression levels in hippocampal homogenates, AD CA1 pyramidal neurons AD CA1 astrocytes and AD CA1 microglia. Bar graph depicts log2 fold change, comparing AD vs. age matched normal controls. No significant difference was detected in homogenates, AD neurons, or AD astrocytes. In stark contrast, a significant four-fold increase (*p<0*.*001*) was observed in AD microglia.

In order to parallel previously published EWAS studies, *ANK1* mRNA expression was initially investigated in homogenate samples derived from human hippocampus. No significant difference in *ANK1* expression was detected between AD and control samples (*p =* .*323*) (Fig 1). These data suggest that the limbic (studied here) and sub-cortical regions (studied in EWAS) may be different in terms of *ANK1* expression, that the number of cells analyzed in homogenates is too large to detect subtle differences in single cell populations, splice variants, or that separate cell populations may demonstrate overlapping and wide variance in *ANK1* expression.

In order to address the effect of cellular heterogeneity, data from laser-captured microglia, astrocytes and neurons were analyzed. Analogous to homogenate samples, no significant difference in *ANK1* expression was found between control neurons and AD neurons (*p>*.*1)*, or control astrocytes and AD astrocytes (*p>*.*1)* (Fig 1). In stark contrast, AD microglia from the same individuals, in the same brain region revealed a significant (*p<0*.*01*) 4.1-fold increase in *ANK1* expression compared to microglia isolated from control brain samples. Quantitation of these findings were replicated using real-time PCR (S2 Fig). These findings suggest that the insignificant change in homogenates between AD and control brain concealed highly significant alterations in *ANK1* expression in microglia in AD. Given that neuronal cells express higher *ANK1* levels compared to microglial samples (Base-Mean 9 vs. 2 respectively) may indicate that the *ANK1* gene may have a significant role in neuronal function although no disease associations can be made. These findings show that expression profiling of laser captured cells is required to clarify data from homogenate studies.

**Fig 2:**
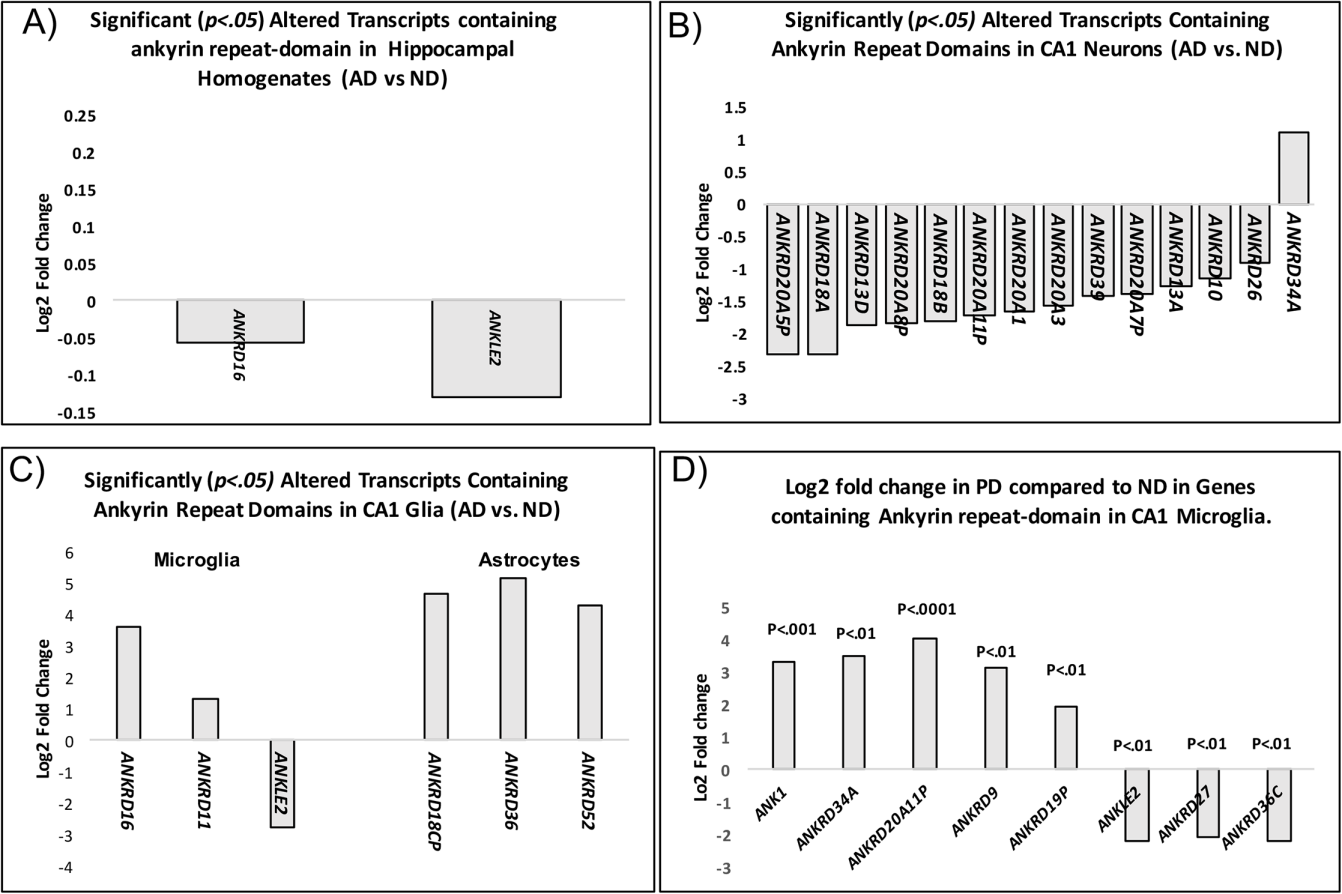
Significant, Log2 fold change(s) in genes containing ankyrin repeat domains in AD hippocampal homogenates, AD CA1 pyramidal neurons, AD CA1 astrocytes and AD CA1 microglia. A) Significantly *(p <* .*05)* altered Ankyrin repeat containing genes in hippocampal homogenates in AD compared to ND. B) Significantly altered (p < .05) Ankyrin repeat containing genes in LCM neurons and glial cells (C). D) average log2 mRNA fold difference in ankyrin repeat genes in PD microglia from CA1 of the hippocampus compared to the same matched control subjects used in AD comparisons. LCM neurons, astrocytes and microglia were derived from the same human subjects. Detailed expression changes can be found in S1 Fig.

In order to determine disease specificity, laser captured CA1 microglia from Parkinson’s cases were also analyzed. Similar differential expression patterns were identified in PD microglia for two of the eight genes analyzed (*ANK1*, *and ANKLE2*) (Fig 2D). These data show that unlike homogenate and LCM neurons, *ANK1* is significantly up-regulated in both AD and PD microglia. These data suggest that alterations in *ANK1*, at least in microglia, may not be disease specific, but rather a response, or phenotype associated with neurodegeneration. Interestingly, when other brain regions were analyzed (e.g. substantia nigra), PD microglia showed a significant 2-fold increase in *ANK1* expression (S1 Table), providing further evidence for the notion that *ANK1* may be involved in, or associated with, the process of neurodegeneration, more specifically, neuroinflammation.

### Analysis of ankyrin repeat-domain genes

In order to determine if other genes containing ankyrin repeat domains were selectively affected by disease, we analyzed all the significantly altered *ANK1*-like genes in homogenates, LCM neurons, astrocytes and microglia.

#### Homogenate samples

Expression data generated from hippocampal homogenates revealed only two significant changes in genes containing ankyrin repeat domains (*ANKRD16 p = <0*.*014) and ANKLE2(p = <* .*0132)* (Fig 2A).

#### Laser captured neurons

Expression data from CA1 neurons show significant changes in fourteen genes containing Ankryin repeat domains (Fig 2B). Thirteen of the fourteen genes were downregulated; the largest fold-change (-2.3) was identified in ANKRD18A (*p = 0*.*0028*), a gene not identified as significantly altered in homogenates, or in LCM glia. The only Ankyrin repeat gene significantly up-regulated was ANKRD34A (*p = 0*.*021*) (Fig 2B).

#### Laser captured microglia

Expression data from laser captured microglia isolated from CA1 of hippocampus show similar expression changes in *ANKLE2 (p = <* .*001)* compared to homogenates (see Fig 2A compared to Fig 2C).

#### Laser capture astrocytes

Expression data from laser captured astrocytes isolated from CA1 of hippocampus from the same individuals showed no overlap in any of the aforementioned ankyrin repeat genes identified in neurons or microglia. Interestingly, three unique ankyrin repeat genes were identified in astrocytes. Significant (*p* < .05) upregulation of ANKRD36 (5-fold increase), ANKRD52 (4.3-fold increase), and ANKRD18CP (4.6-fold increase) were observed. A detailed list of all the Ankyrin repeat genes can be found on S1 Table.

### Differential expression analysis in EWAS related genes

**Fig 3:**
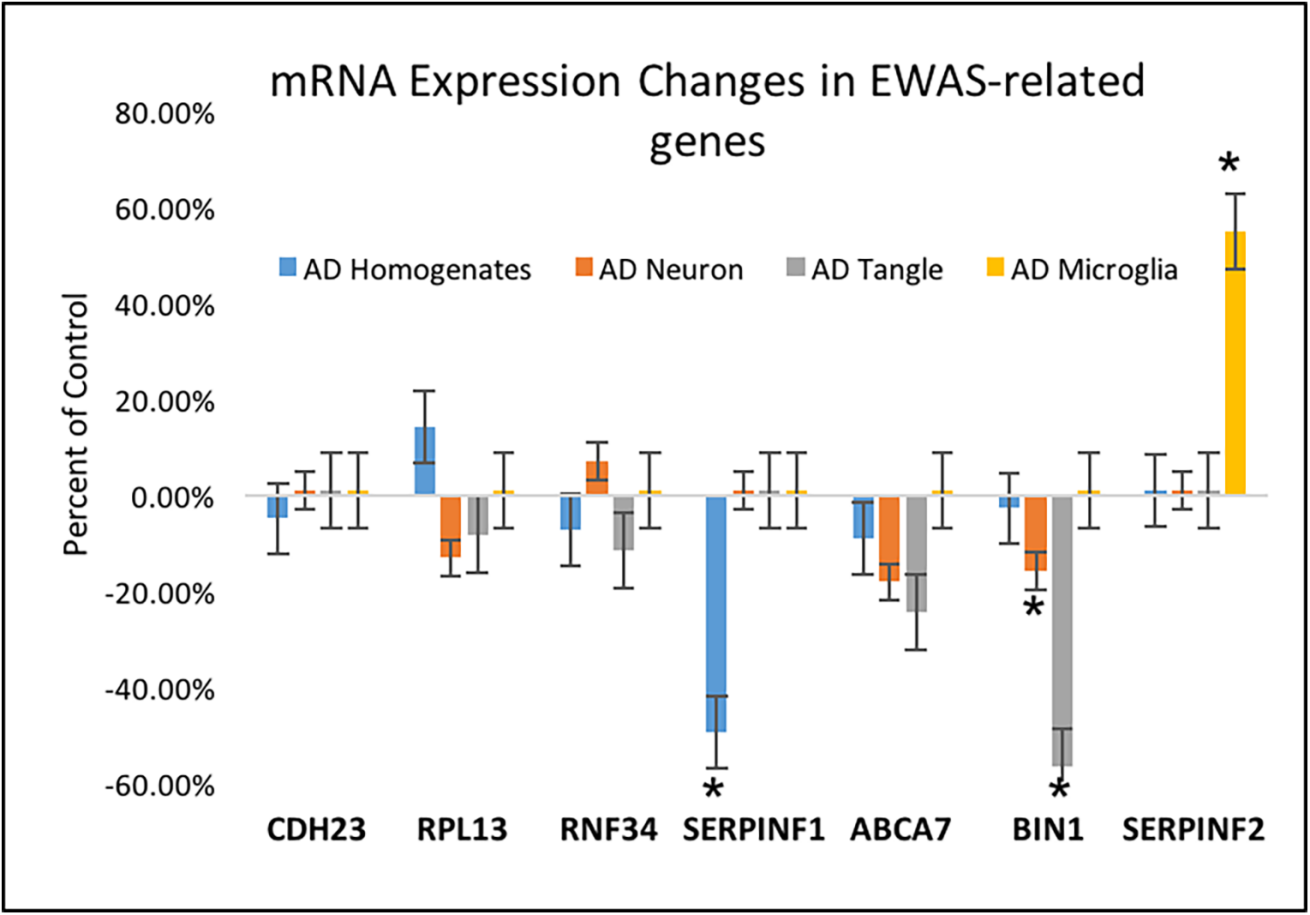
mRNA expression analysis of EWAS-related genes in AD CA1 pyramidal neurons AD CA1 astrocytes and AD CA1 microglia. Only two of the seven identified transcripts in the EWAS study were significantly differentially expressed, *BIN1* in AD neurons and *SERPINF2* in AD microglia. * indicates p < .05.

In addition to the differential methylation changes in the *ANK1* gene in AD [11], recent EWAS studies also identified a number of other loci as being differentially methylated in disease (*ABCA7*, *BIN1*, *CDH23*, *DIP2A*, *RHBDF2*, *RPL13*, *RNF34*, *SERPINF1 and SERPINF2*). Of the nine genes identified by De Jager and colleagues, six were also identified in LCM CA1 neurons (*CDH23*, *RPL13*, *RNF34*, *SERPINF1*, *ABCA7*, *and SERPINF1)*, but only one was found to be significantly differentially expressed Bin 1 (*p = <0*.*021*), (Fig 3).

In microglia, one gene, *SERPINF2*, had a significant *(p = <0*.*001)* increase in expression in AD (*p = <0*.*001*) compared to control (Fig 3). This same gene was not differentially expressed in AD homogenates or AD neurons. These data suggest that homogenates are inadequate predictors of global gene expression changes. In fact, homogenate samples appear to mask important changes in single cell classes.

## Discussion

Recent studies of human brain homogenates have identified hypermethylation of the *ANK1* gene in Alzheimer’s disease[11, 12]. Unfortunately, the interpretation of these data is obscured since brain homogenates produce data that result from many different classes of cells in many different disease states. These authors (DeJager et al., 2014; Lunnon 2014) acknowledge this issue in their homogenate data, but speculate that their *ANK1* findings may be related to activation of microglia through association with *PTK2B*, a gene that is associated with many microglial processes[21–28]. In order to address the issue of cellular heterogeneity, we isolated microglial, astrocytes, and neurons in the same individuals by laser capture microdissection from CA1 of hippocampus in AD, and in normal control cases. Using these data sets, we determined that *ANK1* expression is up-regulated 4-fold in AD microglia, but not in pyramidal neurons or astrocytes from the same individuals.

We note that, although the conventional association between DNA methylation and gene expression is that hypermethylation is repressive, we found increased expression in *ANK1* –the gene that prior studies found to be hypermethylated. We speculate that this apparent inconsistency may be attributable to the location (e.g. intragenic regions) identified in the EWAS study, however other explanations are possible. For example, the data generated in EWAS studies was derived from bisulfite-treated DNA, therefore the signal is the sum of DNA methylation marks including hypermethylation, which may have different effects on transcription. As such, recent studies have shown that an increase in methylation within intragenic regions results in an increase in gene expression and gene activation[29–31].

The lack of altered *ANK1* expression in hippocampal homogenates compared to findings of increased DNA methylation in other studies[11, 12] may be attributed to several factors: 1) the different brain regions studied, for example neocortical vs. limbic in our data; 2) the complexities of the relationship between DNA methylation and gene expression; 3) dilution effects of homogenates and 4) differential splicing of the gene. In addition, the lack of altered *ANK1* expression in hippocampal homogenates in our data, compared to our finding of increased expression of ANK1 in microglia from a hippocampal sub-region, may be attributed to dilution effects of homogenates.

These data show that unlike homogenate and LCM neurons, *ANK1* is significantly up-regulated in both AD and PD microglia, suggesting that alterations in *ANK1*, at least in microglia, may not be disease specific, but rather a response to a common pathway affected in neurodegeneration. (e.g. inflammation, or oxidative stress). The role of increased expression of *ANK1* in Alzheimer’s microglia remains to be determined; but more importantly, these findings emphasize that expression profiling of defined classes of cells is required to clarify data from homogenate studies.

## Supporting information

S1 Table Detailed description, Base Mean expression, log2 fold change, and associated *p-value* of every gene analyzed in this study. (PDF)

S1 Fig Schematic of laser capture microdissection on hippocampal tissue sections (A) Hippocampal sections were immunoreacted using an antibody to LN3 (B), and ~600 LN3 positive microglia cells were captured (C). Individual microglial cells were cut and dropped into an inverted microcentrifuge cap (D) and processed for RNA sequencing or qPCR.
(PDF)

S2 Fig Quantitative RT-PCR of ANK1 gene expression levels in AD CA1 laser capture microglia (green tracer), compared to NC (blue tracers). Note, AD microglial- ANK1 expression levels peak at 25 cycles compared to 30 cycles in NC microglia.
(PDF)
