# Correction: *ANK1* is up-regulated in laser captured microglia in Alzheimer's brain; the importance of addressing cellular heterogeneity

**DOI:** 10.1371/journal.pone.0191382

**Published:** 2018-01-11

**Authors:** Diego Mastroeni, Shobana Sekar, Jennifer Nolz, Elaine Delvaux, Katie Lunnon, Jonathan Mill, Winnie S. Liang, Paul D. Coleman

Fig 3 appears incorrectly in the published article. Please see the correct Fig 3 here.

**Fig 3:**
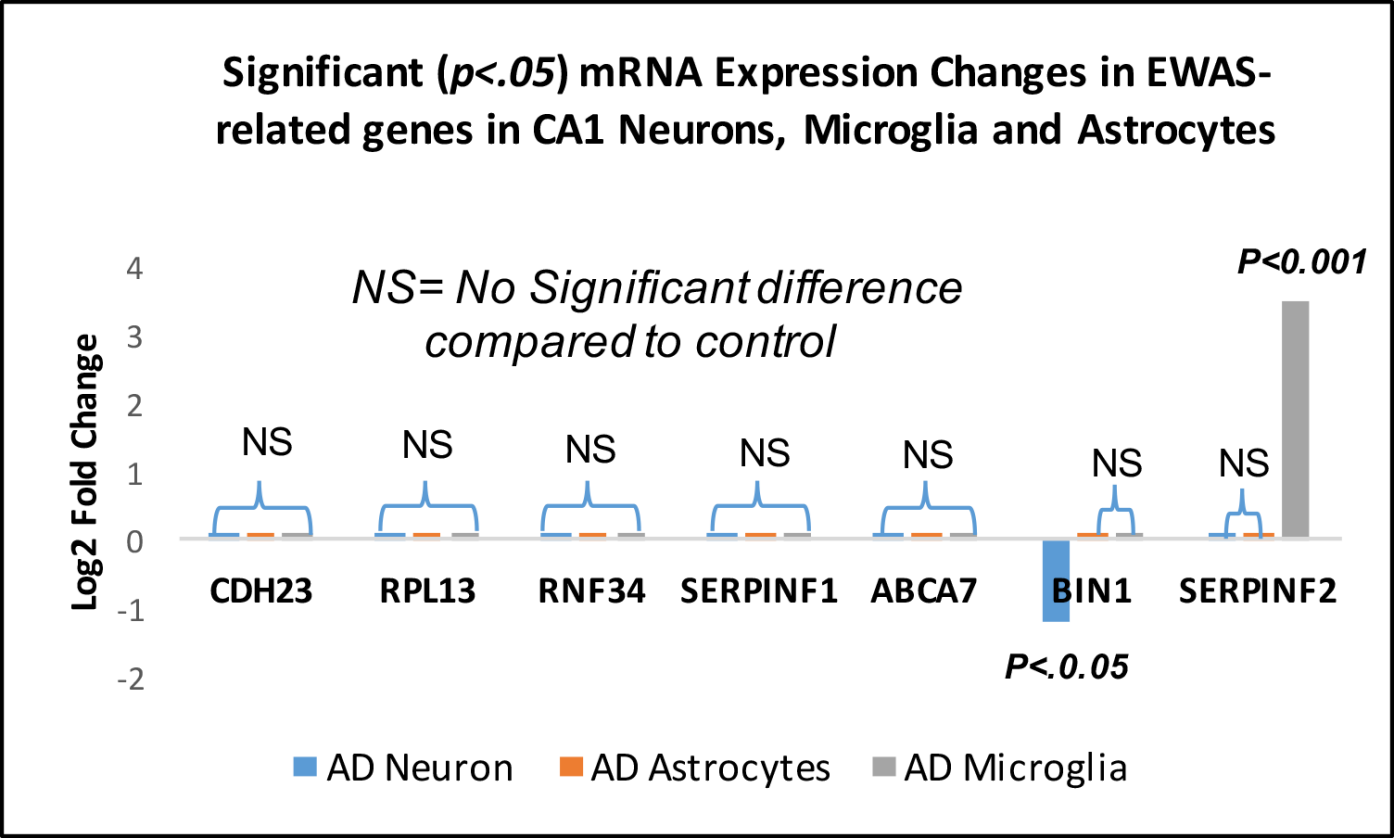
mRNA expression analysis of EWAS-related genes in AD CA1 pyramidal neurons AD CA1 astrocytes and AD CA1 microglia. Only two of the seven identified transcripts in the EWAS study were significantly differentially expressed, BIN1 in AD neurons and SERPINF2 in AD microglia. * indicates p < .05.

There is an error in the third sentence of the Abstract. The correct sentence is: In order to address the issue of cellular heterogeneity in homogenate samples we isolated microglia, astrocytes and neurons by laser capture microdissection from CA1 of hippocampus in the same individuals with a clinical and pathological diagnosis of AD and matched control cases.
