# Supplementary material for: ANK1 is up-regulated in laser captured microglia in Alzheimer’s brain; the importance of addressing cellular heterogeneity
Source: PLoS One. 2017 Jul 12;12(7):e0177814. doi: 10.1371/journal.pone.0177814 (PMC5507536; doi:10.1371/journal.pone.0177814)
Supplement: S1 Table — (PDF) [file pone.0177814.s001.pdf]

Supplementary Figure 1

Cell Type	Gene ID	Associated Gene Symbol	Description	Base Mean	log2FoldChange	*P-value*
AD Microglia						
	ENSG00000029534	ANK1	ankyrin 1, erythrocytic [Source:HGNC Symbol;Acc:492]	1.744955101	4.060125225	0.015363358
	ENSG00000134461	ANKRD16	ankyrin repeat domain 16 [Source:HGNC Symbol;Acc:23471]	2.430756829	3.626557345	0.016607746
	ENSG00000167522	ANKRD11	ankyrin repeat domain 11 [Source:HGNC Symbol;Acc:21316]	105.3712309	1.345186307	0.04569532
	ENSG00000176915	ANKLE2	ankyrin repeat and LEM domain containing 2 [Source:HGNC Symbol;Acc:29101]	30.95356634	-2.789335205	0.006435363
	ENSG00000206560	ANKRD28	ankyrin repeat domain 28 [Source:HGNC Symbol;Acc:29024]	24.70753485	-2.000382048	0.009696268
	ENSG00000187984	ANKRD19P	ankyrin repeat domain 19, pseudogene [Source:HGNC Symbol;Acc:22567]	9.093868298	2.543000749	0.011204671
	ENSG00000167711	SERPINF2	serpin peptidase inhibitor, clade F (alpha-2 antiplasmin, pigment epithelium derived factor), member 2 [Source:HGNC Symbol;Acc:9075]	1.696752291	3.456179299	0.019957356
PD Microglia						
	ENSG00000029534	ANK1	ankyrin 1, erythrocytic [Source:HGNC Symbol;Acc:492]	1.128223296	3.277131583	0.028271004
	ENSG00000176915	ANKLE2	ankyrin repeat and LEM domain containing 2 [Source:HGNC Symbol;Acc:29101]	41.70257426	-2.191991721	0.016692929
	ENSG00000187984	ANKRD19P	ankyrin repeat domain 19, pseudogene [Source:HGNC Symbol;Acc:22567]	7.644783003	1.929043186	0.024533314
	ENSG00000215559	ANKRD20A11P	ankyrin repeat domain 20 family, member A11, pseudogene [Source:HGNC Symbol;Acc:42024]	3.885646501	4.052579451	0.001593965
	ENSG00000236816	ANKRD20A7P	ankyrin repeat domain 20 family, member A7, pseudogene [Source:HGNC Symbol;Acc:31980]	1.44192178	2.83764835	0.042515086
	ENSG00000105186	ANKRD27	ankyrin repeat domain 27 (VPS9 domain) [Source:HGNC Symbol;Acc:25310]	25.30223164	-2.071412197	0.028128904
	ENSG00000181039	ANKRD34A	ankyrin repeat domain 34A [Source:HGNC Symbol;Acc:27639]	11.14374565	3.498821806	0.000610653
	ENSG00000174501	ANKRD36C	ankyrin repeat domain 36C [Source:HGNC Symbol;Acc:32946]	46.96924343	-2.198532374	0.003105954
	ENSG00000156381	ANKRD9	ankyrin repeat domain 9 [Source:HGNC Symbol;Acc:20096]	13.12503529	3.112949826	0.00284704
AD Astrocytes						
	ENSG00000159712	ANKRD18CP	ankyrin repeat domain 18C, pseudogene [Source:HGNC Symbol;Acc:43601]	1.222299433	4.637705359	0.043036902
	ENSG00000135976	ANKRD36	ankyrin repeat domain 36 [Source:HGNC Symbol;Acc:24079]	21.99220833	5.143085664	0.004086619
	ENSG00000139645	ANKRD52	ankyrin repeat domain 52 [Source:HGNC Symbol;Acc:26614]	12.03754509	4.271419728	0.018360323
AD Neurons						
	ENSG00000088448	ANKRD10	ankyrin repeat domain 10 [Source:HGNC Symbol;Acc:20265]	241.5894648	-1.154005335	0.011125185
	ENSG00000076513	ANKRD13A	ankyrin repeat domain 13A [Source:HGNC Symbol;Acc:21268]	124.0582704	-1.265327	0.010618238
	ENSG00000172932	ANKRD13D	ankyrin repeat domain 13 family, member D [Source:HGNC Symbol;Acc:27880]	36.99955169	-1.873516055	0.002596178
	ENSG00000180071	ANKRD18A	ankyrin repeat domain 18A [Source:HGNC Symbol;Acc:23643]	104.471854	-2.334738902	0.002846593
	ENSG00000230453	ANKRD18B	ankyrin repeat domain 18B [Source:HGNC Symbol;Acc:23644]	29.80283082	-1.811576552	0.028989809
	ENSG00000196774	ANKRD20A1	ankyrin repeat domain 20 family, member A1 [Source:HGNC Symbol;Acc:23665]	22.15262523	-1.673717502	0.006363143
	ENSG00000215559	ANKRD20A11P	ankyrin repeat domain 20 family, member A11, pseudogene [Source:HGNC Symbol;Acc:42024]	9.556195895	-1.741233618	0.023628208
	ENSG00000132498	ANKRD20A3	ankyrin repeat domain 20 family, member A3 [Source:HGNC Symbol;Acc:31981]	8.612295194	-1.573272337	0.009469419
	ENSG00000186481	ANKRD20A5P	ankyrin repeat domain 20 family, member A5, pseudogene [Source:HGNC Symbol;Acc:33833]	28.58251991	-2.340679238	0.000282422
	ENSG00000236816	ANKRD20A7P	ankyrin repeat domain 20 family, member A7, pseudogene [Source:HGNC Symbol;Acc:31980]	62.03415887	-1.401833029	0.02961673
	ENSG00000229089	ANKRD20A8P	ankyrin repeat domain 20 family, member A8, pseudogene [Source:HGNC Symbol;Acc:23666]	18.22613082	-1.860830037	0.005505256
	ENSG00000107890	ANKRD26	ankyrin repeat domain 26 [Source:HGNC Symbol;Acc:29186]	245.9087959	-0.916627899	0.027474857
	ENSG00000181039	ANKRD34A	ankyrin repeat domain 34A [Source:HGNC Symbol;Acc:27639]	61.72179588	1.086459643	0.021349041
	ENSG00000029534	ANK1	ankyrin 1, erythrocytic [Source:HGNC Symbol;Acc:492]	9.474936292	-0.265784174	0.750722052
	ENSG00000213337	ANKRD39	ankyrin repeat domain 39 [Source:HGNC Symbol;Acc:28640]	28.39896334	-1.43022794	0.031603889
	ENSG00000136717	BIN1	bridging integrator 1 [Source:HGNC Symbol;Acc:1052]	113.9964078	-1.190540292	0.04532162
