# Supplementary figures and images for: ANK1 is up-regulated in laser captured microglia in Alzheimer’s brain; the importance of addressing cellular heterogeneity
Source: PLoS One. 2017 Jul 12;12(7):e0177814. doi: 10.1371/journal.pone.0177814 (PMC5507536; doi:10.1371/journal.pone.0177814)

Supplementary Figure 1

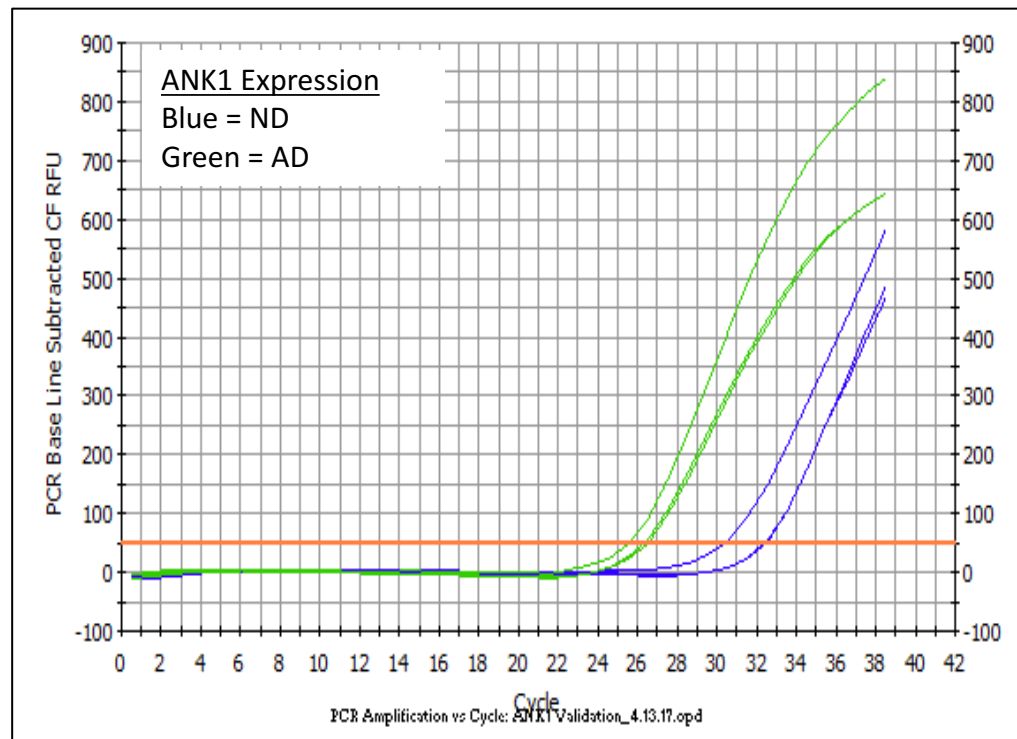

Supplement: S1 Fig — Schematic of laser capture microdissection on hippocampal tissue sections (A) Hippocampal sections were immunoreacted using an antibody to LN3 (B), and ~600 LN3 positive microglia cells were captured (C). Individual microglial cells were cut and dropped into an inverted microcentrifuge cap (D) and processed for RNA sequencing or qPCR. (PDF) [file pone.0177814.s002.pdf]

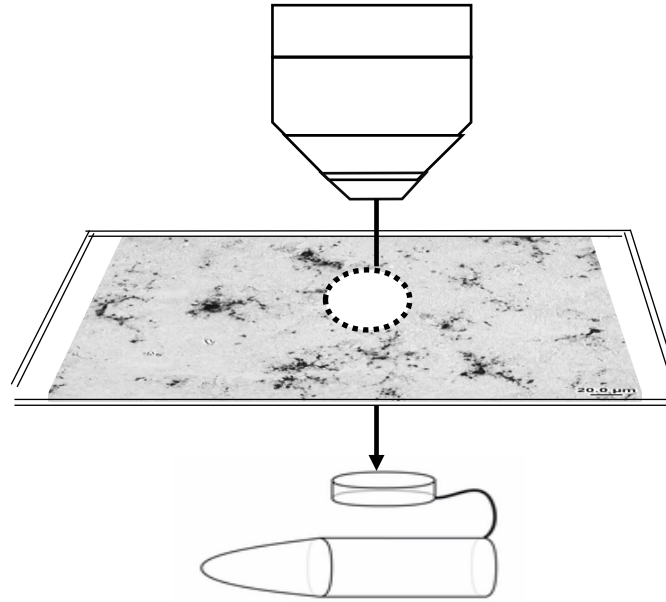

Tissue View pre-cutting 20x

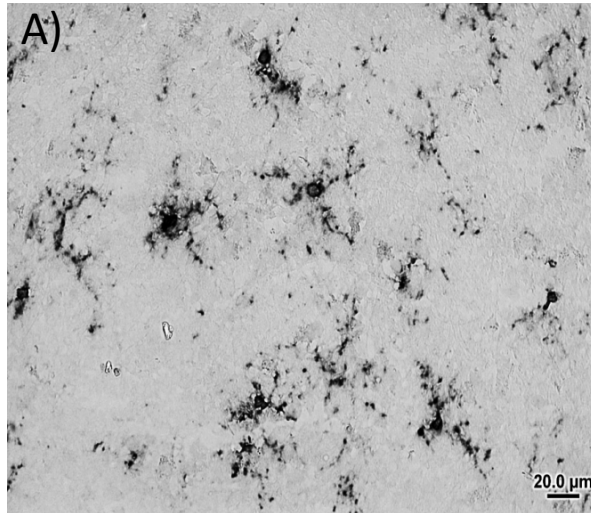

Tissue View After cutting

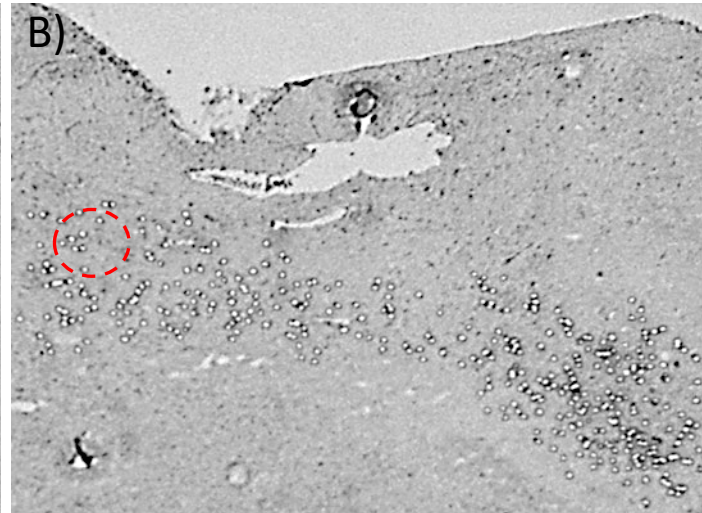

Cap View

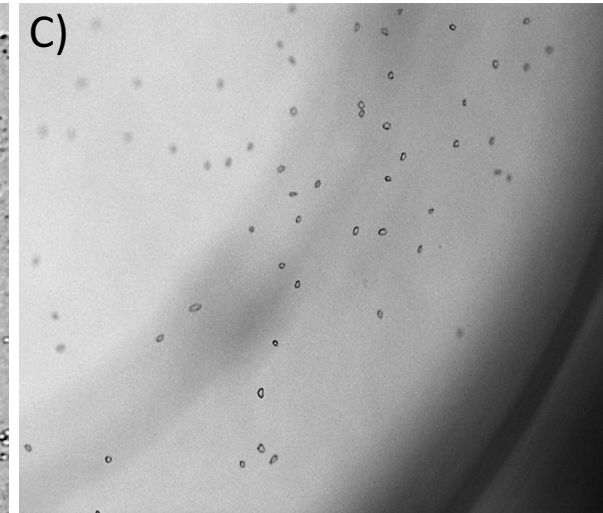

Supplement: S2 Fig — Note, AD microglial- ANK1 expression levels peak at 25 cycles compared to 30 cycles in NC microglia. (PDF) [file pone.0177814.s003.pdf]
